# Evaluation of the Survival of *Lactobacillus fermentum* K73 during the Production of High-Oleic Palm Oil Macroemulsion Powders Using Rotor-Stator Homogenizer and Spray-Drying Technique

**DOI:** 10.3390/microorganisms11061490

**Published:** 2023-06-03

**Authors:** Angélica Clavijo-Romero, Miguel Moyano-Molano, Katherine Bauer Estrada, Lina Vanessa Pachón-Rojas, María Ximena Quintanilla-Carvajal

**Affiliations:** Engineering Department, Universidad de la Sabana, Km 7 vía Autopista Norte, Chía 250001, Colombiakatherinebaues@unisabana.edu.co (K.B.E.);

**Keywords:** high oleic palm oil, *Lactobacillus fermentum*, macroemulsions, probiotics encapsulation, rotor-stator, spray drying

## Abstract

This study aimed to evaluate the survival of the probiotic *Lactobacillus fermentum* when it is encapsulated in powdered macroemulsions to develop a probiotic product with low water activity. For this purpose, the effect of the rotational speed of the rotor-stator and the spray-drying process was assessed on the microorganism survival and physical properties of probiotic high-oleic palm oil (HOPO) emulsions and powders. Two Box–Behnken experimental designs were carried out: in the first one, for the effect of the macro emulsification process, the numerical factors were the amount of HOPO, the velocity of the rotor-stator, and time, while the factors for the second one, the drying process, were the amount of HOPO, inoculum, and the inlet temperature. It was found that the droplet size (ADS) and polydispersity index (PdI) were influenced by HOPO concentration and time, ζ-potential by HOPO concentration and velocity, and creaming index (CI) by speed and time of homogenization. Additionally, HOPO concentration affected bacterial survival; the viability was between 78–99% after emulsion preparation and 83–107% after seven days. The spray-drying process showed a similar viable cell count before and after the drying process, a reduction between 0.04 and 0.8 Log10 CFUg^−1^; the moisture varied between 2.4% and 3.7%, values highly acceptable for probiotic products. We concluded that encapsulation of *L. fermentum* in powdered macroemulsions at the conditions studied is effective in obtaining a functional food from HOPO with optimal physical and probiotic properties according to national legislation (>10^6^ CFU mL^−1^ or g^−1^).

## 1. Introduction

Probiotics are live microorganisms that confer health benefits to the host as long as they reach the site of action in adequate concentration (10^6^–10^9^ CFU mL^−1^ or g^−1^) [1] and have been recognized for their beneficial effects in both humans and animals, playing an essential role in immunological, digestive and respiratory functions [2]. They are mainly members of *Lactobacillus* and *Bifidobacterium* species commonly associated with the human gastrointestinal tract. *Lactobacillus fermentum* K73 is a probiotic strain that shows a hypocholesterolemic effect based on the enzyme bile salt hydrolase (BSH) activity and its cholesterol absorption [3]. However, the inclusion of probiotics in food matrices is still a challenging area of research in food technology [4], as the viable bacterial concentration can decrease due to the processes of integrating the probiotics on different food matrices and the gastrointestinal conditions that they have to survive in order to reach their site of action [5] since the viable bacterial concentration decreases due to the bactericidal effect of gastric juices and bile acids during digestive transit [5].

Emulsification of probiotics and bioactive compounds has been an alternative to solve this problem, conferring protection against environmental and processing conditions and increasing their bioavailability. Usually, emulsification involves two immiscible phases, one of which will be dispersed in the other, with oil and water as the most commonly prepared [6]. There are different techniques to produce emulsions; they differ in the mechanism used to produce micro and nano-droplets of the dispersed phase, such as ultrasound, stirring, high shear homogenization, and high-pressure microfluidization [7]. The use of a rotor-stator for the homogenization of an emulsion has different advantages, such as low cost, easy equipment installation and operation, large volume production, and the ability to manage viscous systems [8]. The emulsion produced by this homogenization should be stable, which depends on the droplet size of the emulsion. To optimize this parameter, the rotational speed is usually modified, as an increase in speed represents a decrease in the droplet size [8,9], in this way producing an effective and stable emulsion [10]. In this sense, HOPO can act as an excellent oil phase for emulsification, as demonstrated by Ricaurte et al., 2016; also, its high nutritional content of β-carotene and vitamin E (between 500–1080 ppm and 110–600 ppm, respectively) and unsaturated fats make this oil one with significant health effects. Due to the *L. fermentum* characteristics and bioactivity, this oil represents an excellent co-encapsulating agent to benefit the survival of this probiotic.

On the other hand, spray drying is one of the most used drying techniques for microencapsulating probiotics on powder presentation [11]. It permits the obtention of powdered probiotics with reduced volume and easier handling, transport, and storage, which implies a reduction in costs [4] as a plus for the stability and protection that conferred against adverse environmental conditions such as oxidation and thermal impacts [3], increasing the survival rate during processing and storage [12]. Its effectiveness is related to the cooling generated by the evaporation of the water, which allows the temperature inside the droplet to remain low, maintaining the characteristics of food products and probiotic survival [13]. However, inlet and outlet temperatures of this process should be controlled, as high temperatures may lead to a loss in the viability of probiotics and degradation of the oil’s thermolabile compounds.

In this way, this study aimed to evaluate the survival of *Lactobacillus fermentum* K73 during the production of high-oleic palm oil macroemulsion powders using a rotor-stator as homogenizer and the spray-drying technique to obtain the powders. The physicochemical characteristics of macroemulsions and powders were also evaluated.

## 2. Materials and Methods

### 2.1. Materials

High oleic palm oil was obtained from Fedepalma (Bogotá, Colombia); whey powder was bought from Alpina (Sopó, Colombia); soy lecithin was bought from Bellchem International (Medellín, Colombia); gelatin was obtained from Tecnoal SAS (Sabaneta, Colombia) and *Lactobacillus fermentum* K73 (GenBank KP784433, NCBI, Bethesda, MD, USA).

### 2.2. Preparation of Cell Culture

*L. fermentum* K73 was activated in Man, Rogosa, and Sharpe (MRS; 1%, *w*/*v*) broth (Scharlau Microbiology, Barcelona, Spain) for 12 h at 37 ± 2 °C (Incubator BD 115L—Binder, Camarillo, CA, USA). The culture medium preparation and the batch fermentation were assessed according to Aragón-Rojas et al. [3] with some modifications. The culture medium pH was adjusted to 5.5, and the fermentation process was kept constant at 37 °C and 100 rpm in a 1 L bioreactor (Bioflo 110, New Brunswick Scientific Co., Inc., Edison, NJ, USA) that had a workload of 0.8 L. The culture medium composition was sweet whey (8% *w*/*v*) and yeast extract (0.2% *w*/*v*). The sweet whey powder had the following composition: protein 11.67% (*w*/*w*), lipid 2.0% (*w*/*w*), lactose 51.64% (*w*/*w*) (AOAC 984.1), and ash 10.9% (*w*/*w*). The reactor was inoculated with *L. fermentum* K73, (10% *v*/*v*), grown in MRS broth with final concentration of approximately 6.27 ± 0.34 Log10 CFU mL^−1^.

### 2.3. Macroemulsion Preparation

#### 2.3.1. Experimental Design

Using the software Design Expert Version 10.1.0 (Stat-Ease Inc., Minneapolis, MN, USA), a Box–Behnken experimental design was carried out by varying three numerical factors: HOPO concentration (1–10%, *w*/*w*), rotational speed (6000–26,000 rpm) and time (1–5 min). Whey (20%, *w*/*w*) and lecithin (10% *w*/*w* concerning the HOPO concentration) concentrations were held constant. In addition, *L. fermentum* K73 was added (10% *v/v*) at a concentration of approximately 10 Log10 CFU mL^−1^ (Table 1).

#### 2.3.2. Preparation of Coarse Emulsions

The coarse emulsions were homogenized in a mixer (Imusa, Bogotá, Colombia), incorporating whey powder (20%, *w*/*w*) followed by the addition of HOPO (proportion indicated by the experimental design) and lecithin (10% *w*/*w* concerning the HOPO concentration) to the distilled water over 1 min. The coarse emulsions and 10% (*v*/***v***) of *L. fermentum* culture (at a concentration of approximately 10 Log10 CFU mL^−1^) were added to the rotor-stator homogenizer (IKA^®^, Magic LAB^®^, Fort Lauderdale, FL, USA) slowly following a Box–Behnken experimental design (Design Expert Version 10.1.0 (Stat-Ease Inc., Minneapolis, MN, USA)) and varying three numerical factors: HOPO concentration (1–10%, *w*/*w*), rotational speed (6000–26,000 rpm) and time (1–5 min). The processing temperature and purity were considered as control criteria. Once the recirculation time was finished, the pH was adjusted to 5.5 with a pH/mV Meter, UltraBASIC, and hydrochloric acid (HCl) 1 N. Finally, the emulsions were distributed in 50 mL sterile falcon tubes.

#### 2.3.3. Macroemulsion Characterization

HOPO macroemulsions were characterized concerning their physical characteristics, such as the average droplet size (ADS), polydispersity index (PdI), zeta potential (ζ), and creaming index (CI). Additionally, the survival of *Lactobacillus fermentum* K73 was evaluated as described below.

##### Droplet Size, Polydispersity Index, and Zeta Potential

The average droplet size (ADS), polydispersity index (PdI), and zeta potential (ζ) were determined using dynamic light scattering equipment (DLS) with a Zetasizer NanoZS laser diffractometer (Malvern Instruments, Malvern, UK) using a water dilution of 1:100 (*v*/*v*). The measurements were performed in duplicate with a dispersion angle of 173° [14,15].

##### Creaming Index

Freshly made macroemulsions were transferred to graduated plastic tubes (50 mL), they were hermetically sealed, and samples were stored for 7 days at two different conditions: room temperature (19 ± 2 °C) and refrigeration temperature (4 ± 2 °C). On day 7, the creaming index was determined as follows:

$$CI=\frac{H_{S}}{H_{E}}\times 100$$

where *H_S_* is the height of the serum, and *H_E_* is the initial height of the macroemulsion [16].

#### 2.3.4. Bacterial Survival

The cell counts of *L. fermentum* K73 after macroemulsion preparation were performed by plate counting in MRS agar after culture at 37 ± 2 °C for 24 h under aerobic conditions [17]. Briefly, 0.1 g of macroemulsion was added to 9.9 mL of peptone water (0.2% *w*/*v*). Then, the serial dilutions were performed, plated on MRS agar, and incubated at 37 ± 2 °C for 24 h. The cell count was expressed as Log10 CFU mL^−1^ [18]. The percentage of survival was calculated by the following equation:

$$(\%)=\frac{{Log\;UFC\;N}_{1}}{Log\;UFC\;N_{0}}\times 100$$

where *N*_1_ represents the total number of viable cells after treatment, and *N*_0_ the initial number of inoculated microorganisms [19].

### 2.4. Powder Obtention

#### 2.4.1. Experimental Design

Using the software Design Expert Version 11.1.0 (Stat-Ease Inc., Minneapolis, MN, USA) a Box–Behnken experimental design was carried out varying three numerical factors: HOPO concentration (1–10% *w*/*w*), bacterial concentration (10–50% *w*/*w*) and inlet temperature (120–175 °C). Whey concentration was kept constant (10% *w*/*w*), and lecithin concentration was held at 10% *w*/*w* with respect to the HOPO concentration (Table 2).

#### 2.4.2. Preparation of Macroemulsion

The coarse emulsions were prepared through a two-step homogenization using a mixer (Imusa, Bogotá, Colombia), incorporating HOPO, lecithin, and the bacteria in the culture medium previously mentioned (Mixture A), and whey powder and water (Mixture B) over 1 min each. Subsequently, Mixture A was homogenized using a rotor-stator (IKA^®^, Magic LAB^®^, Fort Lauderdale, FL, USA) at 2.2 × 10^4^ rpm for 2 min, and then Mixture B was added at 1.0 × 10^4^ rpm for 1 min to obtain the emulsions [20].

#### 2.4.3. Spray Drying

The emulsions were fed into a pilot-scale spray drier (GEA Process Engineering, Mobile MinorTM, GEA Niro, Søborg, Denmark). The equipment was operated with a pneumatic co-current two-fluid nozzle as the atomizer with an orifice diameter of 1 mm; the outlet temperature of drying air was 90 ± 2 °C, and the atomizing air pressure was 0.75 bar. Finally, the powder was collected and placed in polyethylene bags.

#### 2.4.4. Bacterial Survival

The cell count of *L. fermentum* K73 was performed by plate counting technique. Serial dilutions of the powders were prepared at up to 10^–10^ in 0.1% *v/v* peptone water and mixed with vortex for 15 min [3]. From these dilutions, 100 μL was plated on MRS agar and incubated at 37 ± 2 °C for 24 h under aerobic conditions [3]. The cell count was expressed as CFU g^−1^ and then as survival percent. This experiment was undertaken in triplicate.

#### 2.4.5. Physical Properties of Powder

##### Moisture

The moisture content of the flakes was measured from 0.3 g of sample employing an EM 120-HR moisture analyzer at 105 °C (Precisa Gravimetrics AG, Dietikon, Switzerland). Measurements were performed in triplicate.

##### Water Activity (a_w_)

The water activity of the flakes was measured using an AquaLab Series 4 aw meter at 19 °C (Decagon Devices, Inc., Pullman, WA, USA) after the samples were stabilized at 25 °C for 30 min. The measurements were performed in triplicate.

##### Dissolution Rate

The dissolution rate was carried out by adding 2 g of the powders into 50 mL of distilled water [21]. The mixture was agitated in a 100 mL low-form glass beaker with a magnetic stirrer (Heidolph, Schwabach, Germany) at 900 rpm and 17 °C. The time (s) required for the material to dissolve completely was recorded. The measurements were performed in triplicate.

### 2.5. Statistical Analysis

The statistical analysis was performed using the Box–Behnken optimization experimental design methodology in the Design Expert software Version 11 (Stat-Ease Inc., Minneapolis, MN, USA). A statistical significance test was used for the total error criteria with a confidence level of 95%. The significant terms in the model were found through analysis of variance (ANOVA). The fit of the model was evaluated by the R^2^ value.

## 3. Results and Discussion

### 3.1. Effect of Rotor-Stator

Table 1 shows the average of the duplicate or triplicate of the dependent variables. The independent variables include oil concentration, homogenization speed and time, and the dependent variables particle size distribution (ADS), polydispersity index (PdI), zeta potential (ζ), creaming index (CI) and bacterial survival.

#### 3.1.1. Droplet Size (ADS)

**Figure 1:**
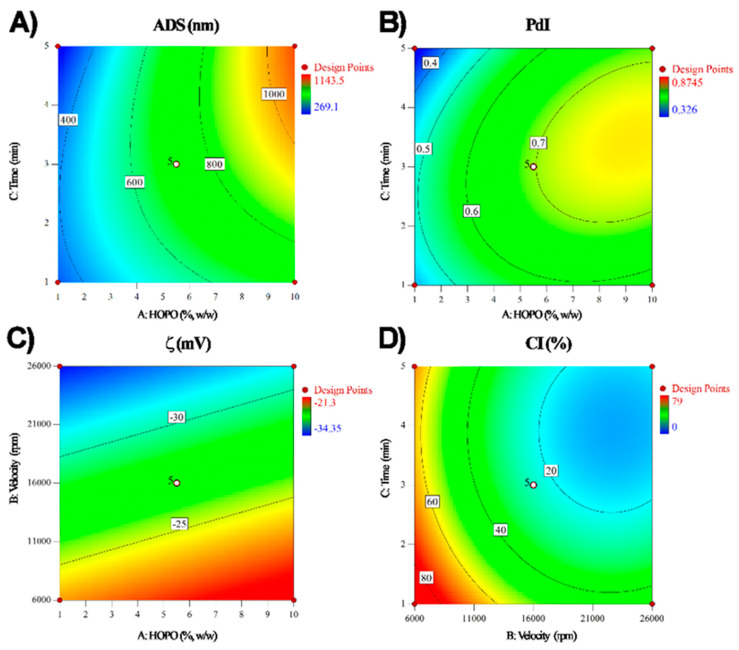
Isoplots for adjusted variables ADS (**A**), PdI (**B**), ζ (**C**), CI (**D**) to the response design.

The values obtained for the droplet size of emulsions were in accordance with the size of *Lactobacillus fermentum*, which is between 0.5 and 0.9 µm. The variance analysis resulted in a quadratic model adjusted with an R^2^ of 0.94 and a non-significant lack of fit (Table 3). The particle size distribution was affected (*p* < 0.05) by the HOPO concentration, time, and some interactions such as HOPO concentration and time (AC) and time squared (B2). From the contour graph for this variable (Figure 1), it was concluded that the relationship between the HOPO concentration and the size was directly proportional. At the same time, the speed did not have a significant effect on the size of the particles. The equations for the prediction of the particle size distribution are shown in Table 4.

At higher HOPO concentrations, the total amounts of oil droplets increase in the emulsion, which directly affects the coalescence rate [22]. Coalescence is a phenomenon caused by the collision of oil droplets; at higher HOPO concentrations, the higher will be the collision of the droplets, which generates a new droplet with a larger size than the initial droplet [23]. HOPO concentration and its interaction with time also affected ADS. This may be caused because, as residence time increases in a rotor-stator homogenization process, shear effort and tension exerted by the plates and their different geometries over the emulsion increase [24]. Break-up mechanisms are present in this process, where the smallest oil particles are detached from the largest particles (erosion), and the agglomerated and aggregates are decreasing (breakage and rupture), hence the particle size [25].

#### 3.1.2. Polydispersity Index (PdI)

From the analysis of variance (ANOVA), a modified quadratic model was obtained that adjusted with a R^2^ of 0.77. Table 3 shows that the model was significant, with a *p*-value lower than 0.05 and a lack of fit greater than 0.05. The factors that significantly affected (*p* < 0.05) this variable were HOPO concentration and time squared (C^2^). From Figure 1, it was concluded that, at lower HOPO concentrations, emulsions with lower polydispersity indices are obtained. The polydispersity index (PdI) is a dimensionless measurement of the distribution of emulsion droplets throughout the phase, with values close to 0 indicating that the sample is monodisperse, and values close to 1 indicating a variety of large droplet sizes [26]. In this case, results showed values between 0.32 and 0.89, which indicated high polydispersity on the samples measured. Since the variation in HOPO concentration is the most common factor that generates recoalescence and aggregation of droplets in emulsion, it is valid to think that those phenomena affect not only the droplet size but also the homogeneity of the distribution of the droplets in the aqueous phase, thus affecting the PdI measurement [27]. The equations for the prediction of the polydispersity index are shown in Table 4. Furthermore, recoalescence of oil droplets decreases as time increases, because the energy applied by the rotor-stator generates the break-up in droplets, making them smaller with the processing time [24], hence the PdI decrease (Figure 1B).

#### 3.1.3. ζ-Potential

ζ-potential is the measurement of the electrostatic charge surrounding the particles. In emulsions, it allows us to estimate their stability [28,29]. In this case, ζ-potential varied between −21.3 and −34.35 mV (Table 1), implying high stability, as values between ±20 and 40 mV provide the system with enough repulsion to promote stability [28]. These values of zeta potential are in accordance with those for emulsions of HOPO obtained by Ricaurte et al., 2018 and Carrion et al., 2021; in these studies, the zeta potential of emulsions was between −14.20 and −40.93 mV, and −30.05 and −42.09 mV, respectively. In these cases, the use of lecithin and whey in the formulation allowed obtaining emulsions charged negatively and with ζ-potential values lower than −30 mV, which are considered stable due to steric and electrostatic repulsion forces between droplets [30,31].

A linear model adjusted with an R^2^ of 0.77 was obtained for this variable. On one hand, Table 2 shows that the model was significant, with a *p*-value lower than 0.05 and a lack of fit greater than 0.05. Results show that the velocity had a significant effect (*p* < 0.05) on the ζ-potential value (Table 2). On the other hand, the HOPO concentration and the time of homogenization did not have a significant effect on it (*p* > 0.05. Table 2). Figure 1C shows the contour plot for this variable. At higher speeds, more negative ζ- potential values were obtained, which translated into greater stability of the emulsions. No scientific papers reported in the literature have studied the effect of agitation speed on the ζ-potential of HOPO macroemulsions obtained using a rotor-stator. Nevertheless, the lower (more negative) ζ-potential values obtained by increasing the homogenization speed in the rotor-stator could be explained by the energy applied by the rotor-stator to the emulsion in the homogenization process [32,33]. The equations for the prediction of the ζ-potential are shown in Table 4.

#### 3.1.4. Creaming Index (CI)

The creaming index measures the destabilization of emulsions by the migration of dispersed droplets to the top of the emulsion driven by the difference in the densities of emulsion phases [34]. Creaming index values at room temperature (19 ± 2 °C) varied between 0 and 78.6% (Table 1). For this variable, a quadratic model with an R^2^ of 0.90 and a non-significant lack of fit (Table 2) was obtained from the analysis of variance (ANOVA), where velocity, time, velocity squared (B^2^), and time squared interactions (C^2^) were significant for the model (*p* < 0.05). In this sense, the increase in homogenization speed and time produced a significant decrease (*p* < 0.05) in the creaming index. This could be related to the lower droplet size obtained by increasing the speed, as smaller particles tend to migrate to the top of the emulsion slower, ensuring longer stability times [32,33,34]. On the contrary, bigger droplet sizes, PdI values, and less negative values of ζ-potential are related to the increase in the possibility of instability processes of the emulsions [9]. These results explain the higher stability (lower CI) found in macroemulsions with small droplet sizes and PdI values.

Figure 1D shows that to obtain emulsions with a low creaming index, high speeds and times of homogenization must be used when using the rotor-stator homogenization process. The equations for the prediction of phase separation are shown in Table 4. On the other hand, adjusting a model for the creaming index at refrigeration temperature was impossible because no significant differences were found between the evaluated emulsions, possibly due to the gelling process accelerated by the low temperature [35].

#### 3.1.5. Bacterial Survival

**Figure 2:**
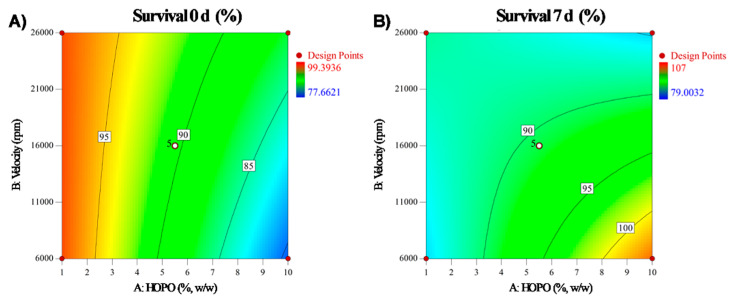
Isoplots for adjusted variable survival (**A**) 0 day (**B**) 7 day.

Both for the bacterial survival at time 0 just after the emulsion preparation and on the seventh day, the analysis of variance (ANOVA) obtained a 2FI model with an R^2^ of 0.8 and a non-significant lack of fit (Table 3). The factors that significantly influenced (*p* < 0.05) bacterial survival were HOPO concentration and HOPO concentration interaction and time (AC) for day 0 and speed time (BC) for day 7. Values were obtained of survival between 78% and 99% for the measurements after the preparation, and between 83% and 101% after seven days of culture, presumably by adaptation and/or cellular recovery of *Lactobacillus fermentum* in the emulsions. For this variable, differences were observed in time according to the contour plot (Figure 2), since for day 0, the greatest viability was obtained with low oil concentration, while at seven days, an opposite behavior was observed since the higher concentration of HOPO increased the viability. The equations for the prediction of phase separation are shown in Table 4.

These results suggest that homogenization in a rotor-stator to form HOPO emulsions with *L. fermentum* under the evaluated conditions does not affect bacterial viability significantly and that it is possible to maintain the cell count for seven days. These results concur with Shimaa et al. [20], who encapsulated *Lactobacillus acidophilus* in O/W/O emulsions using a rotor-stator and concluded that the preparation mode did not affect the viability. Additionally, the difference in survival percentages in relation to HOPO concentrations suggests that the oil plays an important role in protecting *L. fermentum* during processing, as stated by Shimaa et al. [20] and Dowling et al. [36], or that *L. fermentum* may be using the oil as a carbon source during the seven days of incubation. Jo et al. [37] demonstrated that palm oil can significantly promote the growth of *Lactobacillus plantarum* in milk after incubation at 30 °C for two days.

### 3.2. Influence of Spray Drying

According to the results obtained above from the experimental design of *L. fermentum* and HOPO macroemulsions, the drying of these macroemulsions through spray-drying technology was proposed as an encapsulation technique that allows obtaining powders that could have a longer shelf life, preserving the probiotics in better conditions than liquid macroemulsions. For this, an experimental design was carried out with a concentration of HOPO, inoculum, and temperature as factors and viability of probiotic, moisture content, water activity, and dissolution rate of the powders as response variables.

#### Bacterial Survival

For bacterial survival, the analysis of variance (ANOVA) fitted to a quadratic model (0.96 R^2^). Table 5 shows that the model was significant (*p* < 0.05) and had a *p*-value of 0.1794 for the lack of fit, indicating that the proposed model provided a suitable fit and could predict bacterial survival from spray-dried high-oleic palm oil macroemulsions. The factors statistically significant (*p* ≤ 0.05) were the HOPO concentration, the inlet air temperature, the interaction between them, the interaction between bacterial concentration and inlet air temperature, and the quadratic effect of HOPO, as shown in Table 5.

**Figure 3:**
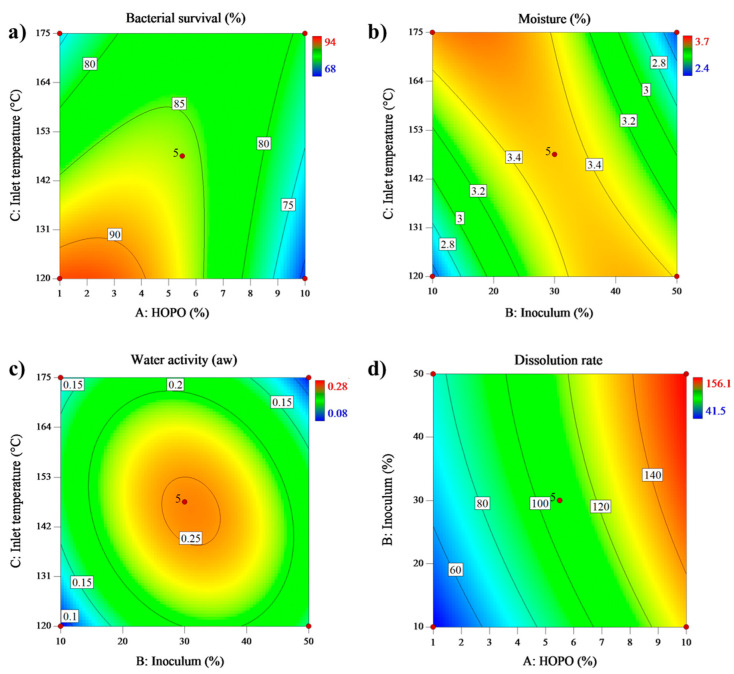
Isoplots for adjusted variables: (**a**) bacterial survival (**b**) moisture (**c**) water activity and (**d**) dissolution rate.

Table 2 showed that the most viable cells could be obtained with HOPO concentration ≤ 5.5 and an inlet air temperature of 147.5 °C, as is reflected in the contour plot (Figure 3), where at low HOPO concentration and inlet air temperature, we found the greatest viability (94%) after emulsification and spray drying.

Table 5 also shows a survival between 68% and 94%, corresponding to 8.28 and 9.93 Log10 CFU g^−1^, the ideal concentration of probiotics for functional food production [38]. Additionally, this concentration would allow the probiotics to arrive at their site of action in a concentration of 10^6^ cells, which is the requirement of the FDA for probiotics after passage through the gastrointestinal tract [1]. The equation obtained for the prediction of bacterial survival is shown in Table 6.

The culture medium proposed by Aragon et al. (2018) was demonstrated to protect the probiotic strains due to the content of whey and yest extract [3]. It can be concluded that the above and the HOPO inclusion in the emulsion protected the microorganisms during emulsification and spray drying, as the microorganisms did not receive the high-shear forces and high temperatures directly, obtaining high survival despite the different formulations and variation in the conditions of the process. These results support the conclusion that emulsification by rotor-stator is an effective technology to preserve the microorganism’s concentration before the application of drying technologies in which the temperature plays an important role in their survival. Both the culture medium and the added whey protect *L. fermentum* K73 from adverse effects such as pressure and temperature during spray drying. The disulfide bonds by the activated protein aggregates act as physical barriers forming a viscous layer on the cell surface, protecting the microorganism from the osmotic stress [3,39].

The results obtained differ from those of other authors that reported a lower survival rate for spray-dried cells, such as Anekella and Orsat (2013) [40], who reported that when using an inlet temperature higher than 130 °C and an outlet temperature between 88–97 °C, the survival of *Lactobacillus acidophilus* and *Lactobacillus rhamnosus* in spray-dried raspberry juice dropped 4.5 Log10 CFU/mL. Likewise, Kingwatee et al. (2015) [41] used maltodextrin and gum arabic in the spray drying of lychee juice with added *Lactobacillus casei*, and the highest outlet temperature (90 °C) showed lower viable cell counts (2.46–4.73 Log10 CFU/g). However, we observed a reduction in the survival of *L. fermentum* K73 between 0.04 and 0.8 Log10 CFU/g using inlet temperatures higher than 130 °C and an outlet temperature of 90 °C.

### 3.3. Physical Properties of the Powders

#### 3.3.1. Moisture

Moisture in a drying process is the ratio between water mass after the process and the total mass of the sample [6]. The results obtained by variance analysis (ANOVA) for the response variable moisture are shown in Table 5. The results were adjusted to a quadratic model with an R^2^ of 0.86. It is observed in Table 5 that the model was significant with a *p*-value lower than 0.05 and a lack of fit > 0.005. This result suggested that the model is adequate and will allow predicting the moisture of HOPO emulsions powders dried in spray drying. Powder moisture varied between 2.4% and 3.7% (Table 2).

The interaction between inlet drying temperature and initial cellular concentration was the variable that significantly affected (*p* < 0.05) the results for moisture. In this way, higher inlet drying temperatures and lower cellular initial concentrations lower the moisture. The equations that describe the behavior of moisture are in Table 6.

Increasing the inlet air temperature generates a decrease in final moisture. That can be attributed to the fact that raising the temperature increases the difference in the temperature gradient between the atomized feed and the drying air, which accelerates the moisture evaporation rate of the sample [42]. A significant gradient also helps to reduce particle size. The smaller the particle, the more enhanced the drying process due to the increased contact area between the hot air flow and the particle’s surface and the increasing distance between the center of the particle and the surface, accelerating the evaporation rate [43]. In addition, its interaction with the initial cellular concentration can explain the moisture increase due to the water present in the initial cellular concentration. The highest initial cell concentration implies a high total water content in the emulsion (90% of the initial cellular concentration is water). The lowest drying temperature (Table 2) cannot evaporate water at the same rate compared with the samples with the lowest cellular initial concentration. Hence, the final moisture will increase [44]. Nevertheless, all results were highly acceptable (moisture < 4%), especially for probiotic products [45]. Powder moisture content strongly influences the product stability and the probiotic viability during storage and is one of the quality parameters to consider in powders containing cells. Moisture content between 4% and 7% is usually recommended for good storage and probiotic viability [46].

Some authors have investigated probiotic drying and the final moisture of the powders; for instance, Arslan-Tontul et al., 2017 [47] studied spray drying of encapsulated *Saccharomyces Bourladii*, *Lactobacillus acidophilus*, and *Bifidobacterium bifidum* using gum arabic as a wall material and its influence on the final powders. They obtained moisture between 6.51% and 8.90%. They concluded that process variables such as inlet temperature, outlet temperature, and flow rate directly affect water evaporation rate and formulation variables such as the type of probiotic. Wall materials are related to their water-binding (hydrophilic or hydrophobic behavior).

#### 3.3.2. Water Activity

Among the parameters that might influence food shelf life, water activity (a_w_) plays an essential role since it reflects the amount of water that is available for chemical reactions and the growth of microorganisms (bacteria, fungi, and yeasts) [48]. Results obtained by analysis of variance (ANOVA) for the response variable water activity are shown in Table 5. The results were adjusted to a quadratic model with an R^2^ of 0.90. It is observed (Table 5) that the model was significant with a *p*-value lower than 0.05 and a lack of fit > 0.005. This result suggests that the model is adequate and hence will allow predicting the water activity of HOPO emulsion powders dried by spray drying.

Cellular initial concentration and inlet drying temperature were the variables that significantly affected (*p* < 0.05) the results for water activity. In this way, higher inlet drying temperatures and higher cellular initial concentrations lower the water activity. The equations that describe the behavior of water activity are in Table 6.

Drying is one of the most efficient ways to conserve foods thanks to, among other parameters, reduced a_w_, which reduces the speed of chemical degradation reactions (among others, non-enzymatic Brownian color, sugar crystallization, and aroma loss) [49], an important aspect to take into consideration in products with antioxidant activity (such as the beta-carotene in the HOPO) and as equal as moisture when an increase in inlet air temperature decreases it, also decreasing the residual water and hence a_w_ values [50]. A lower limit of aw for bacterial growth is around 0.6; this suggests that a higher aw could lead to a reduction in probiotic viability and an increase in the risk of contamination during storage due to the water starting to behave as a solvent that increases the mobility of the products available for microbial growth. However, over-drying may diminish the viability and stability of microorganisms [45].

Some studies suggest that the optimal range of aw values for the storage of probiotics is lower than 0.2, which is the range of the values in this study (0.17 on average). Wang et al., 2004 [43] determined aw during storage, and it can dramatically increase, affecting the bacteria’s viability; spray-dried *Lactobacillus paracasei* CRL 431 was found to survive better when aw was lower than 0.33. However, another study, by Forest et al., 2012 [51] with *L. paracasei* maintained significantly higher survival at an aw of 0.22 (1.2 ± 0.5 × 10^11^ CFU g^−1^) compared with that seen at 0.07 (7.0 ± 1.7 × 10^10^ CFU g^−1^) and 0.33 (2.0 ± 0.4 × 10^10^ CFU g^−1^).

Nevertheless, the survival rate is not related linearly to aw; the relation of water activity and microorganisms in a drying process is complex, combining intrinsic factors of the formulation (nutritive potential, pH, and antimicrobial compounds such as SO_2_, nitrates, and nitrites) and process factors (drying temperature, heat transfer and exposure to oxygen) [52]. Therefore, every study is unique and depends on its factors; in this case, it showed that aw values were based on the viability of the *L. fermentum* K73 emulsions dried in spray drying. However, as cell survival is particularly affected when the food matrix has elevated moisture and water activity (a_w_N0.25) [53], and losses in probiotic viability cause considerable reductions in product shelf life, our results are proposed for the formulation of functional foods with a long shelf life.

#### 3.3.3. Dissolution Rate (DR)

The dissolution rate for spray drying was expressed as the time it takes to solubilize a sample of powder in water until a homogeneous solution without suspended particles is attained (dissolution rate). This variable adjusts to a quadratic model with an R^2^ of 0.99. It is observed (Table 5) that the model was significant with a *p*-value lower than 0.05 and a lack of fit > 0.005. This result suggests that the model is adequate and hence will allow predicting the dissolution rate of HOPO emulsion powders dried in spray drying.

Table 5 shows that all of the variables and their interactions significantly affect the dissolution rate; at higher inlet drying temperature, initial cellular concentration, and lower HOPO concentration, a lower dissolution rate is obtained. No studies (at least to our knowledge) directly relate spray-dried emulsions with microorganisms and the dissolution rate of their powders; however, in the study, the dissolution rate is a crucial aspect to consider for the capacity of powders to rehydrate. The influence of temperature on the hydration of probiotic powders in drying is still under study. Authors such as Perdana et al. [54] discuss how slow drying kinetics (4 mL min^−1^) under relatively low temperatures (between 40 °C and 120 °C) led to significant difficulty in rehydration in *Lactobacillus plantarum*, hence viability loss.

Increasing the HOPO concentration of 1% to 10% decreases the dissolution rate from 60 s to 150 s. Table 2 shows that an increase in HOPO concentration implies an increase in droplet size in the fresh emulsion, and allows to obtain droplet sizes so large that their dispersion is reduced due to the phenomenon of coalescence and flocculation associated with the whey needed to encapsulate HOPO and the homogenization in the rotor-stator [54]. When emulsions with those larger droplets pass through the spray drying process, they generate larger droplet powders with more non-encapsulated HOPO, which decreases the powder dissolution rate. The effect of formulation in fresh emulsions dried over the dissolution rate and the hydration capacity of their powders is highly related to functionalities such as metabolic activity, tolerance toward human gastrointestinal juices, adherence to epithelial surfaces, antagonistic activity against pathogens, and immunoregulatory capacities [44].

Some studies evaluated different conditions that can improve the rehydration of spray-dried microorganisms, such as the study by Goibier and collaborators [22], which dried *Streptococcus thermophilus* and *Bifidobacterium longum* and varied the amount of probiotic powder per mL of water and the temperature of rehydration, resulting in an enhanced recovery of live cells steadily in line with the rehydration temperature (3 × 10^9^ CFU g^−1^, 3.5 × 10^9^ CFU g^−1^, 5.2 × 10^9^ CFU g^−1^, and 6.1 × 10 CFU g^−1^ at 10 °C, 15 °C, 20 °C and 25 °C, respectively).

Besides the studies mentioned above, there are no studies on the dissolution rate of microorganisms encapsulated in emulsions and dried in spray drying for food matrix (all studies were focused on pharmacology and drug delivery); hence this work is highly interesting for food science and probiotics and is recommended to deepen the field.

## 4. Conclusions

The preparation of HOPO macroemulsions in a rotor-stator did not affect the viability of *L. fermentum*, which makes this technology an excellent alternative for producing functional foods that guarantee the action of probiotic bacteria at the appropriate concentrations. Furthermore, high homogenization speeds in the rotor-stator produced the smaller droplet size, the lowest PdI and CI, and the most stable (most negative) ζ-potential values (<−30 mV) under the evaluated conditions.

Spray drying of emulsions of HOPO with probiotics is a promising technology for obtaining powders with the inclusion of *L. fermentum* K73 as an alternative for the development of functional foods with optimal physical and probiotic properties, quality standards, nutritional requirements, and cost reduction. High survival of *L. fermentum* K73 was obtained despite using higher inlet and outlet temperatures that other authors postulated could be lethal for the probiotic cells.

The results obtained were in the range to obtain a product with a prolonged shelf life due to low moisture and aw results. The best formulation for the highest survival corresponds to 5.5% *w*/*w* of HOPO, a homogenization speed of 1.0 × 104 rpm, an inlet temperature of 147.5 °C and 30% *w*/*w* of inoculum, with a yield of 70% powder.

## 5. Patents

This study has a patent CO2021013445A1.

## Figures and Tables

**Table 1:** Response optimization surface experimental design methodology for preparation of macroemulsions and variables adjusted to the model: ADS, PdI, ζ, creaming index, and bacteria survival.

Run	HOPO	Velocity	Time	ADS	PdI	ζ	CI ro	CI rf	Bacterial	Bacterial
	(% *w*/*w*)	(rpm)	(min)	(nm)		(mV)	(%)	(%)	Survival 0 *	Survival 7 *
	[A]	[B]	[C]						(%)	(%)
1	5.5	26,000	1	517.8	0.50	−33.2	43	54	99	99
2	5.5	26,000	5	469.2	0.35	−32.5	32	13	96	85
3	5.5	6000	5	610.9	0.59	−21.5	71	0	83	99
4	5.5	16,000	3	602.9	0.53	−29	21	0	95	89
5	5.5	6000	1	412.5	0.48	−21.3	79	14	97	84
6	1	16,000	5	269.1	0.32	−30.8	0	0	91	86
7	1	26,000	3	295.3	0.42	−31	0	0	94	83
8	5.5	16,000	3	690.9	0.60	−29	36	0	97	84
9	1	16,000	1	287.2	0.38	−27.6	57	0	98	92
10	5.5	16,000	3	850.1	0.71	−28.3	21	0	88	92
11	5.5	16,000	3	691.2	0.75	−30	21	0	89	88
12	1	6000	3	279	0.38	−23.5	79	0	99	89
13	10	6000	3	733.6	0.66	−21.3	57	0	80	107
14	10	26,000	3	899.1	0.59	−34.3	21	0	81	80
15	10	16,000	5	1144	0.72	−21.3	29	14	86	94
16	5.5	16,000	3	745	0.87	−24.4	21	0	89	90
17	10	16,000	1	668.7	0.54	−23.5	57	7	78	101

* Days.

**Table 2:** Response Box–Behnken experimental design for adjusted variables to the model: moisture, aw, dissolution rate and bacteria survival.

Run	HOPO (% *w*/*w*) [A]	Inoculum (% *w*/*w*) [B]	Inlet Temperature (°C) [C]	Moisture (%)	a_w_	Dissolution Rate (s)	Bacterial Survival (%)
1	1	30	175	3.1	0.13	62.4	76
2	1	30	120	3.3	0.18	139.1	78
3	1	50	147.5	3.3	0.27	108.4	88
4	10	30	175	3.4	0.18	151.8	68
5	5.5	50	175	3.7	0.27	106.8	86
6	5.5	30	147.5	3.5	0.28	107.4	87
7	10	30	120	3.3	0.27	107.9	88
8	5.5	30	147.5	3.4	0.16	120.4	91
9	5.5	30	147.5	3.2	0.15	60.33	94
10	5.5	10	175	2.8	0.16	156.1	73
11	10	10	147.5	3.4	0.18	108.4	84
12	10	50	147.5	3.3	0.12	41.5	81
13	5.5	30	147.5	3.5	0.12	85.6	92
14	5.5	30	147.5	2.8	0.12	74.7	82
15	1	10	147.5	2.6	0.18	109.5	74
16	5.5	50	120	2.8	0.15	132.5	78
17	5.5	10	120	2.4	0.12	84.2	81

**Table 3:** ANOVA for the adjusted variables to response optimization design: ADS, PdI, ζ, creaming index and bacterial survival.

	ADS (nm)			PdI			ζ (mV)			CI ro (%)			Survival 0 * (%)			Survival 7 * (%)		
	SS	df	*p*-Value	SS	df	*p*-Value	SS	df	*p*-Value	SS	df	*p*-Value	SS	df	*p*-Value	SS	df	*p*-Value
Model	9.14 × 10^8^	9	0.0014	4.64	9	0.0107	255.56	3	0.0002	8975.94	9	0.0099	496	6	0.0306	594.75	6	0.0309
A-HOPO	6.70 × 10^8^	1	<0.0001	2.29	1	0.001	19.53	1	0.089	98	1	0.4428	136.1	1	0.0312	128	1	0.0511
B-Velocity	2639.01	1	0.5819	0.11	1	0.2697	235.99	1	<0.0001	4512.5	1	0.0009	0	1	0.8824	128	1	0.0511
C-Time	46,003.03	1	0.0468	0.023	1	0.6028	0.038	1	0.9368	1352	1	0.0194	78.13	1	0.087	18	1	0.4255
AB	5561.43	1	0.4298	0.046	1	0.467				462.25	1	0.1207	100	1	0.0574	110.25	1	0.0668
AC	60,737.6	1	0.0278	0.21	1	0.1439				210.25	1	0.2724	156.3	1	0.0229	0.25	1	0.9239
BC	15,246.08	1	0.208	0.39	1	0.0621				2.25	1	0.9054	25	1	0.3083	210.25	1	0.0176
A2	5880.07	1	0.4175	0.45	1	0.0485				29.01	1	0.6714						
B2	67,785.97	1	0.0222	0.23	1	0.1301				1345.33	1	0.0196						
C2	31,518.78	1	0.0863	0.76	1	0.017				870.07	1	0.0459						
Lack of Fit	22,606.94	3	0.5086	0.24	3	0.4601	56.48	9	0.4141	857	3	0.0531	190.1	6	0.7772			0.0906
Pure Error	32,860.89	4		0.31	4		18.67	4		180	4		26.8	4				
R^2^	0.94			0.89			0.77			0.89			0.8			0.8		
R^2^ adjusted	0.86			0.75			0.72			0.76			0.61			0.61		

* Days.

**Table 4:** Equations for ADS, PdI, ζ, creaming index and bacterial survival.

Equations for	
ADS (nm) =	−205.03609 + 30.25170 × A + 0.047122 × B + 141.78146 × C + 8.28611 × 10^−4^ × A × B + 13.69167 × A × C − 3.08687 × 10^−3^ × B × C − 1.84543 × A2 − 1.26883 × 10^−6^ × B2 − 21.63000 × C2
1/PdI =	+4.42968 − 0.25659 × A − 1.22762 × 10^−4^ × B − 0.71806 × C + 2.39322 × 10^−6^ * A × B − 0.025602 × A × C + 1.55251 × 10^−5^ × B × C + 0.016082 × A2 + 2.34043 × 10^−9^ × B2 + 0.10624 × C2
ζ (mV) =	−20.33424 + 0.34722 × A − 5.43125 × 10^−4^ × B − 0.034375 × C
CI amb (%) =	+183.91856 − 4.03519 × A − 9.29639 × 10^−3^ × B − 31.89306 × C + 2.38889 × 10^−4^ × A × B + 0.80556 × A × C − 3.75000 × 10^−5^ × B × C − 0.12963 × A2 + 1.78750 × 10^−7^ × B2 + 3.59375 × C2
Bacterial survival 0 (%) =	+112.30645 − 4.77778 × A − 2.61111 × 10^−4^ × B − 3.38194 × C + 1.11111 × 10^−4^ × A × B + 0.69444 × A × C − 1.25000 × 10^−4^ × B × C
Bacterial survival 7 (%) =	+66.34199 + 2.83889 × A + 1.32917 × 10^−3^ × B + 5.20278 × C − 1.16667 × 10^−4^ × A × B − 0.027778 × A × C − 3.62500 × 10^−4^ × B × C

**Table 5:** ANOVA for the adjusted variables to response Box–Behnken experimental design: bacterial survival, moisture, aw and dissolution rate.

	Bacterial Survival			Moisture			Water Activity			Dissolution Rate		
	SS	df	*p*-Value	SS	df	*p*-Value	SS	df	*p*-Value	SS	df	*p*-Value
Model	821.3	9	0.0004	1.77	9	0.026	0.0616	9	0.0099	16,536.3	9	<0.0001
A-HOPO	162.0	1	0.0006	0.001	1	0.866	0.0028	1	0.1397	14,498.5	1	<0.0001
B-Initial cell count	18.00	1	0.0908	0.020	1	0.502	0.0003	1	0.5960	1708.20	1	<0.0001
C-Inlet temperature	32.00	1	0.0347	0.001	1	0.866	0.0004	1	0.5266	50.65	1	<0.0001
AB	9.00	1	0.2083	0.062	1	0.258	0.0000	1	0.8796	23.04	1	0.0011
AC	196.0	1	0.0003	0.000	1	1.000	0.0001	1	0.7626	54.54	1	<0.0001
BC	196.0	1	0.0003	0.902	1	0.002	0.0036	1	0.1015	37.82	1	0.0002
A^2^	186.2	1	0.0004	0.060	1	0.265	0.0047	1	0.0693	10.10	1	0.0095
B^2^	11.46	1	0.1618	0.656	1	0.005	0.0292	1	0.0010	106.58	1	<0.0001
C^2^	1.78	1	0.5573	0.020	1	0.502	0.0155	1	0.0058	33.57	1	0.0003
Lack of fit	22.00	3	0.1794	0.177	3	0.241	0.0002	3	0.9905	3.76	3	0.1846
Pure error	10.80	4		0.112	4		0.0069	4		1.89	4	
R^2^	0.961			0.859			0.896			0.999		
R^2^—Adjusted	0.912			0.678			0.763			00.999		

**Table 6:** Equations for bacterial survival, moisture, water activity and dissolution rate.

Equations for	
Bacterial survival (%) =	+57.27295 − 5.31552 × A + 2.13222 × B + 0.257444 × C − 0.016029 × A × B + 0.055544 × A × C − 0.012744 × B × C − 0.307500 × A² − 0.004108 × B² − 0.000812 × C²
Moisture (%) =	−3.25651 + 0.029759 × A + 0.176768 × B + 0.053889 × C + 0.001283 × A × B − 0.000039 × A × C − 0.000861 × B × C − 0.005931 × A² − 0.000988 × B² − 0.000093 × C²
Water activity =	−1.32034 + 0.034325 × A + 0.010850 × B + 0.017657 × C − 0.002742 × A² − 0.000169 × B² − 0.000060 × C²
Dissolution rate =	−96.20644 + 17.80039 × A + 2.41291 × B + 1.26607 × C − 0.025340 × A × B − 0.028196 × A × C − 0.005445 × B × C − 0.310556 × A² − 0.012446 × B² − 0.003544 × C²

## Data Availability

Data will be available by request.
